# Baicalein inhibits IL-1β- and TNF-α-induced inflammatory cytokine production from human mast cells via regulation of the NF-κB pathway

**DOI:** 10.1186/1476-7961-5-5

**Published:** 2007-11-26

**Authors:** Chia-Jung Hsieh, Kenton Hall, Tuanzhu Ha, Chuanfu Li, Guha Krishnaswamy, David S Chi

**Affiliations:** 1 Departments of Internal Medicine, James H. Quillen College of Medicine, East Tennessee State University, Johnson City, Tennessee 37614, USA; 2 Departmen of Surgery, James H. Quillen College of Medicine, East Tennessee State University, Johnson City, Tennessee 37614, USA

## Abstract

**Background:**

Human mast cells are multifunctional cells capable of a wide variety of inflammatory responses. Baicalein (BAI), isolated from the traditional Chinese herbal medicine Huangqin (*Scutellaria baicalensis Georgi*), has been shown to have anti-inflammatory effects. We examined its effects and mechanisms on the expression of inflammatory cytokines in an IL-1β- and TNF-α-activated human mast cell line, HMC-1.

**Methods:**

HMC-1 cells were stimulated either with IL-1β (10 ng/ml) or TNF-α (100 U/ml) in the presence or absence of BAI. We assessed the expression of IL-6, IL-8, and MCP-1 by ELISA and RT-PCR, NF-κB activation by electrophoretic mobility shift assay (EMSA), and IκBα activation by Western blot.

**Results:**

BAI (1.8 to 30 μM) significantly inhibited production of IL-6, IL-8, and MCP-1 in a dose-dependent manner in IL-1β-activated HMC-1. BAI (30 μM) also significantly inhibited production of IL-6, IL-8, and MCP-1 in TNF-α-activated HMC-1. Inhibitory effects appear to involve the NF-κB pathway. BAI inhibited NF-κB activation in IL-1β- and TNF-α-activated HMC-1. Furthermore, BAI increased cytoplasmic IκBα proteins in IL-1β- and TNF-α-activated HMC-1.

**Conclusion:**

Our results showed that BAI inhibited the production of inflammatory cytokines through inhibition of NF-κB activation and IκBα phosphorylation and degradation in human mast cells. This inhibitory effect of BAI on the expression of inflammatory cytokines suggests its usefulness in the development of novel anti-inflammatory therapies.

## Background

Human mast cells are multifunctional cells involved in numerous immune and inflammatory reactions [1,2]. Mast cells have been implicated in acute and chronic inflammatory responses and in many diseases characterized by inflammation [3]. The fact that mast cells accumulate at sites of inflammation, such as the nasal mucosa of patients with allergic rhinitis [4], the lung smooth muscle of patients with asthma [5], the skin of patients with urticaria [6], and the joints of patients with arthritis [7], illustrates the association of mast cells in these inflammatory diseases [8]. Our previous reviews have summarized the important role mast cells play in allergic, asthmatic, and inflammatory responses, conditions caused by the production of mediators and select inflammatory cytokines [1,2].

Interleukin-6 (IL-6), interleukin-8 (IL-8), and monocyte chemotactic protein 1 (MCP-1) are important inflammatory cytokines that are secreted from activated mast cells. IL-6 is a multifunctional protein. In innate immunity, it stimulates the synthesis of acute-phase proteins by hepatocytes and thus contributes to the systemic effects of inflammation [9]. In adaptive immunity, it stimulates the growth of B cells that have differentiated into antibody producers [10]. IL-8 is a potent neutrophil chemotactic and activating factor. It serves as a chemical signal that attracts neutrophils to the site of inflammation [11]. MCP-1 is a member of the CC subgroup of the chemokine superfamily [12]. MCP-1 is known for its ability to act as a potent chemoattractant and activator of monocytes/macrophages [13,14]. IL-1β is secreted mainly by macrophages. IL-1β is produced in response to various stimulants, such as bacteria, viruses, and cytokines [15]. Tumor necrosis factor-alpha (TNF-α) is a cytokine involved in systemic inflammation and is a member of a group of cytokines that stimulate the acute phase reaction [16,17]. Our previous studies have shown that IL-1β and TNF-α activated human mast cells to produce selected inflammatory cytokines [18,19]

Baicalein (BAI) is a flavonoid originally isolated from the roots of the traditional Chinese herbal medicine Huangqin, *Scutellaria baicalensis *Georgi. It has been widely employed for many centuries in the traditional Chinese herbal medicine as popular antibacterial, antiviral, and anti-inflammatory agents [20]. Historically, *Scutellaria baicalensis *has been used to treat respiratory tract infection, diarrhea, jaundice, and hepatitis. Recent investigations showed it had broad anti-inflammatory activities. BAI suppressed the LPS-induced production of NO in RAW 264.7 mouse macrophages [21]. It has shown to have potent neuroprotective effect on LPS-induced injury of dopaminergic neurons [22]. Recently, BAI has been shown to inhibit inflammation through inhibition of COX-2 gene expression [23] and to suppress LPS induced degradation of IκBα and activation of NF-κB [24]. However, the molecular effects of BAI on inflammatory cytokine expression by human mast cells had not been studied.

The purpose of this study is to investigate effects and mechanisms of BAI on inflammatory cytokine expressions from IL-1β- and TNF-α-activated human mast cells. Our results showed that BAI inhibited the production of inflammatory cytokines through inhibition of NF-κB activation and IκBα phosphorylation and degradation in human mast cells. This inhibitory effect of BAI on the expression of inflammatory cytokines suggests its usefulness in the development of novel anti-inflammatory therapies.

## Methods

### Reagents and cells

**Figure 1:**
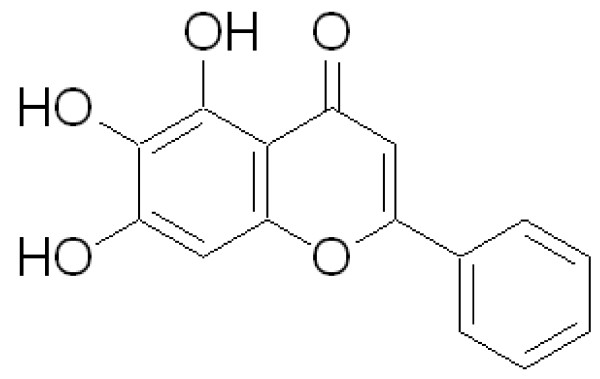
Structure of Baicalein.

The baicalein (Fig. 1) was purchased from Sigma (St. Louis, MO). HMC-1 cell line, established from a patient with mast cell leukemia, was graciously provided by Dr. Joseph H. Butterfield (Mayo Clinic, Rochester, MN). IL-1β, TNF-α, and ELISA kits of IL-6, IL-8, and MCP-1 were purchased from R&D (Minneapolis, MN). RPMI 1640 media and HEPES were obtained from GibcoBRL (Rockville, MD). 2-mercaptoethanol was purchased from Sigma (St. Louis, MO). Fetal bovine serum was obtained from Atlanta Biologicals (Atlanta, GA). RNA-BEE was purchased from Tel-Test, Inc. (Friendswood, Texas). Gene Amp RNA PCR Core Kit was purchased from Applied Biosystems (Branchburg, NJ).

### Cell culture

HMC-1 cells were cultured and maintained in RPMI 1640 media with 5 × 10^-5 ^2-mercaptoethanol, 10 mM HEPES, gentamycin 50 μg/ml, 5 μg/ml insulin, transferrin and sodium selenite, 2 mM L-glutamine, and 5% heat inactivated fetal bovine serum in a 37°C incubator with 5% CO_2_. The cell cultures were maintained in 75 cm^2 ^flasks (Corning) [25].

### Induction of cytokine production

Two ml of HMC-1 mast cells at 1 × 10^6 ^cells/ml concentration were cultured with or without various concentrations of BAI in the presence or absence of IL-1β (10 ng/ml) or TNF-α (100 U/ml) for 24 hrs [18]. The cultures were carried out in triplicate. At the end of incubation, supernatants were harvested for measuring IL-6, IL-8, and MCP-1 by ELISA, and cell viability and numbers of the culture were analyzed. The cell viability was determined by trypan blue dye exclusion. Trypan blue dye (0.4%) was added to cell samples in a ratio of 1:2.5 and preparations were viewed with a standard light microscope [18]. The ratio of live to dead cells (cell viability) was determined. The cell viabilities of the drug groups in this study were ranging from 93 to 95%, while that of medium control cultures was 93%. BAI, IL-1β, or TNF-α at the concentrations used in this study appeared to have no toxic effect to the HMC-1 cultures.

### ELISA for cytokine production

Cytokine ELISA was performed for IL-6, IL-8, and MCP-1. ELISA was carried out on cell-free culture supernatants using commercially available ELISA kits, according to manufacturer's instructions as earlier described. Results were analyzed on an ELISA plate reader (Dynatech MR 5000 with supporting software) [18].

### Analysis of cytokine gene expression by RT-PCR

HMC-1 were treated with the appropriate reagents and allowed to incubate at 37°C with 5% CO_2 _for 6 hours before being harvested for RNA. RNA was extracted from HMC-1 (3 × 10^6 ^cells) by the addition of 1 ml of RNA-BEE. After the addition of chloroform and shaking for 1 minute the samples were centrifuged at 12,000 × g for 15 minutes at 4°C to achieve phase separation. Isopropanol was added to the aqueous phase, and the preparation was frozen at -20°C overnight. The following day, the samples were centrifuged at 12,000 × g for 30 minutes at 4°C. The RNA pellet was washed with 1 ml 75% ethanol containing DEPC and allowed to air dry. The pellet was resuspended in DEPC water and quantitated by optical density readings at 260 nm. Reverse Transcriptase Polymer Chain Reaction (RT-PCR) was performed with a Gene Amp RNA PCR Core Kit according to manufacturer's instructions. cDNA was synthesized with murine leukemia virus reverse transcriptase (2.5 U/μl), 10× PCR buffer (500 mM KCl, 100 mM Tris-HCl, pH 8.3), 1 mM each of the nucleotides dATP, dCTP, dGTP and dTTP; RNase inhibitor (1 U/μl), MgCl_2 _(5 mM), and oligo(dT)_16 _(2.5 μM) as a primer. The samples were incubated at 42°C for 20 minutes, 99°C for 20 minutes, and 5°C for 5 minutes in a DNA thermocycler (Perkin-Elmer Corp., Norwalk, CT) for reverse transcription. PCR of cDNA was done with MgCl_2 _(1.8 mM), each of the dNTPs (0.2 mM), AmpliTaq polymerase (1 U/50 μl), and paired cytokine-specific primers (0.2 nM of each primer) to a total volume of 50 μl. Cycles consisted of 1 cycle of 95°C for 2 min, 35 cycles of 95°C for 45 sec, 60°C for 45 sec, and 72°C for 1 min 30 sec, and lastly, 1 cycle of 72°C for 10 min. Ten microliters of the sample were electrophoresed on a 2% agarose gel and stained with ethidium bromide for viewing. Primer sequences used are as follows: HPRT: 5' CGA GAT GTG ATG AAG GAG ATG G 3' and 5' GGA TTA TAC TGC CTG ACC AAG G 3'; IL-6: 5' ATG AAC TCC TTC TCC ACA AGC GC 3' and 5' GAA GAG CCC TCA GGC TGG ACT G 3'; IL-8: 5' ATG ACT TCC AAG CTG GCC GTG GCT 3' and 5' TCT CAG CCC TCT TCA AAA ACT TCT C 3'; and MCP-1: 5' GTA GAA CTG TGG TTC AAG AGG 3' and 5' AGC CAC CTT CAT TCC CCA AG 3'. Densitometry was done by normalizing target genes to house keepers using Un-Scan-It Version 5.1 software (Orem, UT).

### NF-κB assay in HMC-1

HMC-1 were stimulated with PMA, IL-1β, TNF-α, and/or BAI for 24 hours, and then harvested for electrophoretic mobility shift assay (EMSA) [26-29]. Cells were washed with PBS and mixed with one hundred microliters of hypotonic buffer which contains: 10 mM HEPES pH 7.9, 10 mM KCl, 0.1 mM EDTA, 0.1 mM EGTA, 1 mM dithiothreitol (DTT), 0.5 mM phenylmethylsulfonyl fluoride (PMSF), 1 μM aprotinin, 1 μM pepstatin, 14 μM leupeptin, 50 mM NaF, 30 mM β-glycerophosphate, 1 mM Na_3_VO_4_, and 20 mM p-nitrophenyl phosphate. Cells were incubated over ice for 30 minutes and then vortexed after the addition of 6.25 μl of 10% of Nonidet P-40. After 2 minutes of centrifugation at 30,000 × g, supernatants were kept at -80°C while the pellets were collected and vortexed every 20 minutes for 3 hours in 60 ml of a hypertonic salt solution: 20 mM HEPES pH 7.9, 0.4 M NaCl, 1 mM EDTA, 1 mM EGTA, 12 mM DTT, 1 mM PMSF, 1 μM aprotinin, 1 μM pepstatin, 14 μM leupeptin, 50 mM NaF, 30 mM β-glycerophosphate, 1 mM Na_3_VO_4_, and 20 mM p-nitrophenyl phosphate. Nuclear translocation of NF-κB was analyzed by the EMSA using the nuclear fraction. Seven micrograms of nuclear protein were added to 2 ml of binding buffer (Promega, Madison, WI), and 35 fmol of double stranded NF-κB consensus oligonucleotide (5' AGT TGA GGG GAC TTT CCC AGG C 3') (Promega, Madison, WI) end labeled with γ-P32 ATP (Amersham Biosciences, Piscataway, NJ). The samples were incubated at room temperature for 20 minutes and run on a 5% nondenaturing polyacrylamide gel for 2 hours. A supershift assay using antibodies to P65 and P50 was performed to confirm NF-κB binding specificity as previously described [26-29].

### Western blot analysis for IκBα

Cytoplasmic proteins (40 μg) were mixed with 2× SDS sample buffer, heated at 95°C for 5 min, and separated by SDS-polyacrylamide (12.5%) gel electrophoresis [27,30]. The separated proteins were transferred onto Hybond enhanced chemiluminescence membranes (Amersham) and then incubated with an appropriate rabbit primary antibody [IκBα antibody (Santa Cruz Biotechnology) or phosphorylated IκBα antibody (New England Biolabs)] in Tris-buffered saline – 0.05% Tween 20 containing 5% nonfat dry milk for 1 – 2 hours at room temperature. After they were washed three times in Tris-buffered saline – 0.05% Tween 20, the membranes were incubated with peroxidase-conjugated goat anti-rabbit Ig G (Sigma Chemical) for 1 hour at room temperature. After three washes in PBS, the conjugated peroxidase was visualized by enhanced chemiluminescence according to the manufacturer's instructions (Amersham). The protein signals of IκBα were quantified by scanning densitometry (Genomic Solutions).

### Statistical analysis of the data

All experiments were done in triplicate. The data were analyzed by Student's two-tailed *t*-test using Statistica software (StatSoft, Inc., Tulsa, OK). All data were reported as means ± SE. A *p*-value of less than 0.05 was considered significant.

## Results

### BAI inhibits IL-1β- and TNF-α-induced IL-6, IL-8, and MCP-1 production in mast cells

**Figure 2:**
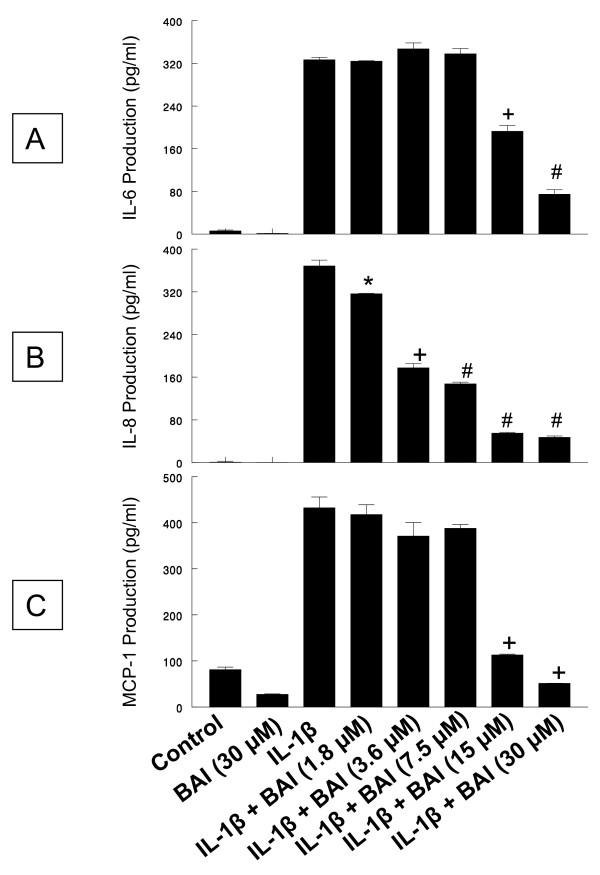
**Effects of Baicalein (BAI) on production of IL-6, IL-8, and MCP-1 from IL-1β-activated HMC-1 cells**. To each well of a 6-well culture plate, two ml of HMC-1 (1 × 10^6 ^cells/ml) were cultured alone (Control), or in the presence of BAI (30 μM), IL-1β (10 ng/ml), and the combinations of IL-1β (10 ng/ml) with different concentrations of BAI (1.8 to 30 μM) for 24 hrs in triplicate. Supernatants were harvested for measuring IL-6, IL-8, and MCP-1 by ELISA. The IL-6 (Panel A), IL-8 (Panel B), and MCP-1 (Panel C) production was significantly decreased when BAI was added in IL-1β-activated HMC-1 cells. *, +, and # indicate p < 0.05, <0.0005, and <0.00005, respectively, when compared with the IL-1β-treated group.

First, the effect of BAI on production of the inflammatory cytokines, IL-6, IL-8, and MCP-1, from IL-1β- and TNF-α-activated HMC-1 cells was studied. BAI at concentrations of 1.8, 3.6, 7.5, 15, and 30 μM have been proved to be non-toxic to HMC-1 [31]. Two mL of HMC-1 at 1 × 10^6 ^cells/mL were cultured with the above mentioned concentrations of BAI in the presence or absence of IL-1β (10 ng/mL) for 24 hrs. The cell free supernatants were collected and assayed for cytokines by ELISA. The results are shown in Fig. 2. IL-1β at 10 ng/mL concentration markedly induced IL-6, IL-8, and MCP-1 production from HMC-1 (326.7 ± 8.0, 368.1 ± 19.1, and 432.4 ± 40.9 pg/mL, respectively). BAI alone did not induce cytokine production from HMC-1. However, BAI at 15 and 30 μM concentrations significantly decreased the IL-1 β-induced IL-6 production to 192.7 ± 18.7 and 74.6 ± 14.6 pg/mL, respectively (p < 0.0005 and p < 0.00005, respectively) and MCP-1 production to 112.9 ± 3.1 and 51.2 ± 0.5 pg/mL, respectively (both p < 0.0005). BAI at all tested concentrations (1.8 to 30 μM) significantly decreased the IL-1 β-induced IL-8 production, in a dose-dependent manner, to 316.4 ± 1.3, 177.4 ± 13.2, 147.6 ± 5.4, 54.9 ± 3.3, and 46.9 ± 4.4 pg/mL, respectively (p < 0.05 for 1.8 μM, p < 0.0005 for 3.6 μM, and p < 0.00005 for all the rest).

**Figure 3:**
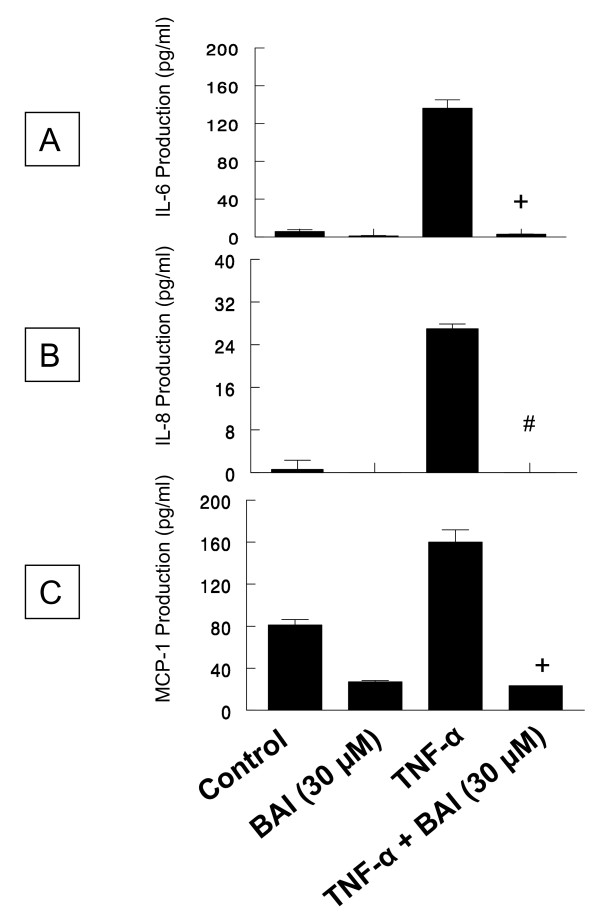
**Effects of Baicalein (BAI) on production of IL-6, IL-8, and MCP-1 from TNF-α-activated HMC-1 cells**. To each well of a 6-well culture plate, two ml of HMC-1 (1 × 10^6 ^cells/ml) were cultured alone (Control), or in the presence of BAI (30 μM), TNF-α (100 U/ml), and the combinations of TNF-α (100 U/ml) with BAI (30 μM) for 24 hrs in triplicate. Supernatants were harvested for measuring IL-6, IL-8, and MCP-1 by ELISA. The IL-6(Panel A), IL-8 (Panel B), and MCP-1 (Panel C) production was significantly decreased when BAI was added in TNF-α-activated HMC-1. + and # indicate p <0.0005 and <0.00005, respectively, when compared with the TNF-α-treated group.

TNF-α also activated HMC-1 to product inflammatory cytokines, but to a lesser extent (136.2 ± 15.4 pg/mL for IL-6, 27.0 ± 1.5 pg/mL for IL-8, and 160 ± 20.4 pg/mL for MCP-1). Since BAI at 30 μM was the most effective concentration in inhibition of cytokine production in IL-1β-activated HMC-1, we decided to only use this concentration in experiments with TNF-α-activated HMC-1. Similarly, BAI at 30 μM concentration has been shown to significantly decrease the TNF-α-induced production of IL-6, IL-8, and MCP-1 to 3.0 ± 0.3, 0.0 ± 0.0, and 23.4 ± 0.23 pg/mL, respectively (p < 0.00005 for IL-8 and p < 0.0005 for the rest) (Fig. 3).

### Effects of BAI on IL-6, IL-8, and MCP-1 gene expressions in activated mast cells

**Figure 4:**
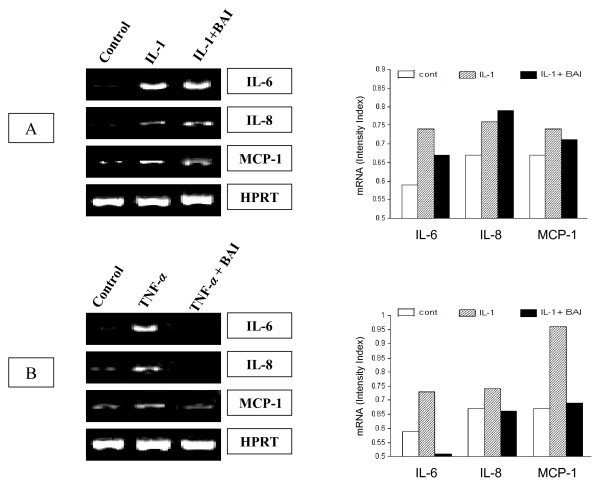
**RT-PCR analysis of effects of BAI on the gene expression of IL-6, IL-8, and MCP-1 in IL-1β- and TNF-α-activated HMC-1 cells**. HMC-1 cells were treated with: A. IL-1β (10 ng/ml) with and without BAI (30 μM), and B. TNF-α (100 U/ml) with and without BAI (30 μM) for 6 hours before harvested for RNA preparation. RNA was subjected to RT-PCR with specific primers for target genes. HPRT was used as a house keeping gene to ensure equal loading. There were mild decreased gene expressions in IL-6 and MCP-1 and mild increased in IL-8 by BAI co-cultured with IL-1β-activated HMC-1 (Panel A). However, markedly decreased gene expressions of IL-6, IL-8, and MCP-1 were showed by BAI co-cultured with TNF-α-activated HMC-1 (Panel B). By densitometric analysis, the ratio of the expression of cytokine to HPRT was calculated and assigned as the intensity index as shown in the bar graph.

To study effects of BAI on inflammatory cytokine gene expression, the experiments were performed using IL-1β- and TNF-α-activated HMC-1. HMC-1 were treated with IL-1β or TNF-α in the presence or absence of BAI (30 μM) for 6 hours and harvested for transcriptional analysis via RT-PCR. IL-1β-treated HMC-1 increased IL-6, IL-8, and MCP-1 mRNA transcription (Fig. 4A). The intensities of the cytokine and house keeping gene (HPRT) bands were measured by densitometry, and the ratio of the cytokine to the house keeping gene was calculated and assigned as the intensity index. In the presence of BAI, the expression of IL-6 and MCP-1 was slightly decreased, while IL-8 faintly increased. The intensity indices for IL-6 expression were 0.74 and 0.67 for the IL-1β and the IL-1β plus BAI groups, respectively. The intensity indices for IL-8 expression were 0.76 and 0.79 for the IL-1β and the IL-1β plus BAI groups, respectively, while that for MCP-1 expression were 0.74 and 0.71 for the IL-1β and the IL-1β plus BAI groups, respectively.

In TNF-α-activated HMC-1, BAI markedly decreased the inflammatory cytokine gene expression (Fig. 4B). The intensity index for IL-6, IL-8, and MCP-1 expression in TNF-α-activated HMC-1 were 0.73, 0.74, and 0.96, respectively. When HMC-1 cells were activated by TNF-α in the presence of BAI (30 μM), the intensity index for IL-6, IL-8, and MCP-1 were decreased to 0.51, 0.66, and 0.69, respectively.

### Role of NF-kB activation in the inhibitory effect of BAI on inflammatory cytokine production from IL-1β- and TNF-α-activated mast cells

**Figure 5:**
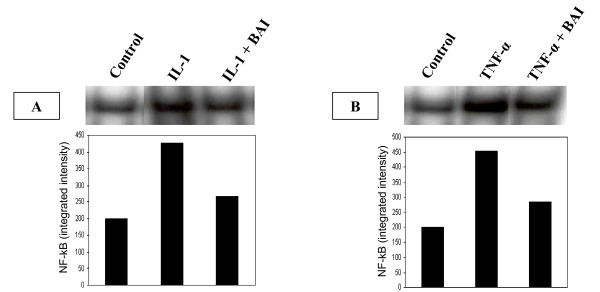
**Effects of BAI on NF-κB translocation in IL-1β- and TNF-α-activated HMC-1 cells**. HMC-1 cells were cultured with IL-1β or TNF-α in the presence or absence of BAI (30 μM) for 24 hours. NF-κB translocation was analyzed by a shift in oligonucleotide binding in EMSA gels. NF-κB translocation was decreased by BAI co-cultured with IL-1β (panel A) and TNF-α (panel B) when compared with the IL-1β or TNF-α alone. Densitometric analysis of NF-κB was expressed as integrated intensity and shown in the bar graph.

NF-κB is an important transcription factor that mediates the transcription of many proinflammmatory cytokine genes [32,33]. In order to study the role that NF-κB plays in the inhibitory effect of BAI on inflammatory cytokine production, NF-κB activation was analyzed in HMC-1 cultured with IL-1β or TNF-α in the presence or absence of BAI (30 μM). In the presence of BAI, NF-κB translocation, as seen by a shift in oligonucleotide binding in EMSA gels, was decreased in the IL-1β- (Fig. 5A) and TNF-α-activated HMC-1 (Fig. 5B).

### Role of IkBα proteins in the inhibitory effect of BAI on inflammatory cytokine production from IL-1β- and TNF-α-activated mast cells

**Figure 6:**
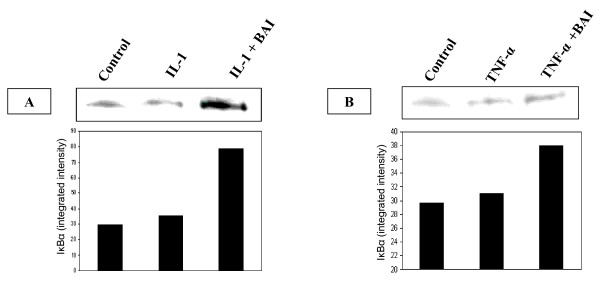
**Effects of BAI on IκBα proteins levels in cytoplasm of IL-1β- and TNF-α-activated HMC-1 cells**. HMC-1 cells were cultured with IL-1β or TNF-α in the presence or absence of BAI (30 μM) for 24 hours. Cytoplasmic extracts were prepared from each sample, and levels of IκBα proteins were analyzed by Western blot. BAI co-cultured with IL-1β (panel A) and TNF-α (panel B) showed markedly increased intensities when compared with the IL-1β or TNF-α alone. Densitometric analysis of IκBα was expressed as integrated intensity and shown in the bar graph.

The activation of NF-κB requires phosphorylation and proteolytic degradation of the inhibitory protein IκBα [34]. To determine whether the inhibitory activity of BAI is due to its effect on IκBα phosphorylation and degradation, we used Western blot analysis to examine the cytoplasmic levels of IκBα in HMC-1 after treatment with IL-1β or TNF-α in the presence or absence of BAI (30 μM). The data showed that in the presence of BAI, the IκBα protein levels were markedly increased in the IL-1β- (Fig. 6A) and TNF-α-activated HMC-1 (Fig. 6B).

## Discussion

Inflammatory cytokines are important factors in chronic inflammation, allergy, asthma, atherogenesis, and autoimmune diseases. Human mast cells play an integral role in the inflammatory response by accumulating at sites of inflammation and mediating the production of inflammatory cytokines [35]. In spite of advances in the pharmacological management of above mentioned diseases and symptoms, to discover effective, alternative anti-inflammatory reagents is still in need. Several Chinese herbal medicines have anti-bacterial and viral properties and been used for treatment of chronic inflammation. Previously, we have screened several Chinese herbal medicines and found that the compound Baicalein (BAI, Fig 1) isolated from Huangqin (*Scutellaria baicalensis *Georgi) has a great inhibitory effect on the production of IL-6 from IL-1β-activated HMC-1 in a dose dependent fashion [31]. The purpose of this study is to further investigate inhibitory effects and mechanisms of BAI on inflammatory cytokine expression from IL-1β- and TNF-α-activated human mast cells. Ultimately it is hoped that BAI will be a possible candidate for future development of novel anti-inflammatory therapies.

In this study, we examined effects of BAI on the production of important inflammatory cytokines, IL-6, IL-8, and MCP-1, from IL-1β- or TNF-α-activated HMC-1. We observed that BAI (1.8 to 30 μM) significantly inhibited production of IL-6, IL-8, and MCP-1 in a dose-dependent manner in IL-1β-activated HMC-1 (Fig. 2). Since BAI 30 μM was the most effective concentration, we only used this dose to treat TNF-α-activated HMC-1 cells and found it also significantly inhibited production of IL-6, IL-8, and MCP-1 in TNF-α-activated HMC-1 (Fig. 3). The results show that BAI significantly inhibit the production of inflammatory cytokines from human mast cells. The cell viabilities of the drug groups in this study were ranging from 93 to 95%, while that of medium control cultures was 93% (Data not shown). Thus, this inhibitory effect appears not due to the toxic effect of BAI on HMC-1 cells. Moreover, the gene expression, analyzed by RT-PCR, of these inflammatory cytokines was mildly decreased in IL-1β-activated HMC-1 (Fig. 4A) and markedly decreased in TNF-α-activated HMC-1 (Fig. 4B) when BAI was presented. These suggest that inhibitory effect of BAI on cytokine productions is through the decrease of cytokine mRNA transcription.

BAI is a flavonoid extracted from the root of Scutellaria baicalensis Georgi, which has been used as anti-inflammatory medicine in China for years. In recent studies, an important flavonoid, quercetin, has been reported to exert a strong inhibitory effect on the production of IL-6, MCP-1, and histidine decarboxylase (HDC) mRNA transcription from mast cells [36-38]. Our results confirmed that BAI, as a flavonoid, could also strongly inhibit production of inflammatory cytokines of IL-6, IL-8, and MCP-1 from activated mast cells through the decrease of mRNA transcription. On the other hand, in our study, the cytokine gene expression was mildly decreased in IL-1β-activated HMC-1 (Fig. 4A), but markedly decreased in TNF-α-activated HMC-1 (Fig. 4B) by addition of BAI. It appears that BAI had a differential effect on the cytokine gene expression in mast cells activated by different stimulants. It has been shown that acute phase response cytokines, IL-1β and TNF-α, activate human mast cells by IL-1 receptor (IL-1R) and TNF-α receptor (TNFR) signaling pathways, respectively, involving MyD88 dependent and/or independent protein kinases [39,40]. This differential effect of BAI on activated mast cells warrants further studies.

The expression of various inflammatory cytokines is regulated by transcription factors. The activation of the NF-κB transcription plays an important role in inflammation through its ability to induce the transcription of proinflammatory genes [41]. Previously, glucocorticoids that have frequently been used for the treatment of inflammatory diseases, allergy, and autoimmune diseases were suggested to suppress NF-κB activation. Glucocorticoids are thought to induce the transcription of IκBα, resulting in an enlarged IκBα pool, and therefore reduced active NF-κB in the nucleus [42]. Additionally, 12-lipoxygenase (12-LOX) has been implicated as a mediator of inflammation, atherosclerosis, and cancer [43-45]. Several *in vitro *studies have suggested 12/15-LOX products to be co-activators of peroxisomal proliferator activating-receptors (PPAR), regulators of cytokine generation, and modulators of gene expression related to inflammation resolution. The dampening effect of PPAR on inflammation is via their inhibitory activity on expression of NF-κB [46-48]. As BAI is known as a 12-LOX inhibitor, we speculated the mechanism by which BAI inhibited inflammatory cytokines was through the NF-κB/IκBα pathway. Therefore, we analyzed NF-κB activation and examined the cytoplasmic levels of IκBα in HMC-1 after treatment with IL-1β or TNF-α in the presence or absence of BAI. Our data showed BAI decreased NF-κB binding activity (Fig. 5) and increased IκBα proteins in cytoplasm in IL-1β- and TNF-α-activated mast cells (Fig. 6). The results suggest BAI inhibits the NF-κB activation via inhibition of IκBα phosphorylation and degradation.

## Conclusion

In searching for effective drugs to treat inflammatory related diseases, we found baicalein from the Chinese herbal medicine possesses strong inhibitory effect on production of selected inflammatory cytokines from human mast cells. The inhibitory mechanism appears to be due to inhibition of NF-κB activation pathway and IκBα phosphorylation and degradation. This inhibitory effect of baicalein on the expression of inflammatory cytokines indicates its usefulness in the development of novel anti-inflammatory therapies.

## List of abbreviations

BAI, Baicalein

EMSA, electrophoretic mobility shift assay

HMC-1, human mast cell-1

IκBα, inhibitor of κB alpha

MCP-1, monocyte chemotactic protein 1

NF-κB, nuclear factor-kappa B

## Competing interests

The author(s) declare that they have no competing interests.

## Authors' contributions

CJH conducted experiments, participated in the experimental design, and wrote the manuscript. KH conducted experiments. TH and CL contributed to the experiments of EMSA and Western blot. GK oversaw research. DSC conceived of the study, contributed to the experimental design and coordination, and edited the manuscript. The authors have had the opportunities to both read and revise the manuscript.
